# Supplementary material for: Pyruvate Dehydrogenase Kinase 1 inhibition mediated oxidative phosphorylation enhancement in cartilage promotes osteoarthritis progression
Source: BMC Musculoskelet Disord. 2023 Jul 20;24:597. doi: 10.1186/s12891-023-06585-6 (PMC10357736; doi:10.1186/s12891-023-06585-6)
Supplement: Supplementary file 1 — Additional file 1. [file 12891_2023_6585_MOESM1_ESM.zip › BP.Gsea.1653623667859/GOBP_GLYCOSYLATION.html]

Details for gene set GOBP\_GLYCOSYLATION[GSEA]

|  || Dataset | 55457\_collapsed\_to\_symbols.cls.cls #OA\_versus\_normal.cls.cls #OA\_versus\_normal\_repos |
| Phenotype | cls.cls#OA\_versus\_normal\_repos |
| Upregulated in class | OA |
| GeneSet | GOBP\_GLYCOSYLATION |
| Enrichment Score (ES) | 0.31085098 |
| Normalized Enrichment Score (NES) | 1.5688225 |
| Nominal p-value | 0.0 |
| FDR q-value | 0.2937405 |
| FWER p-Value | 1.0 |
Table: GSEA Results Summary

  

Fig 1: Enrichment plot: GOBP\_GLYCOSYLATION      
 Profile of the Running ES Score & Positions of GeneSet Members on the Rank Ordered List

  

| SYMBOL | TITLE | RANK IN GENE LIST | RANK METRIC SCORE | RUNNING ES | CORE ENRICHMENT || 1 | C1GALT1C1 | C1GALT1 specific chaperone 1 [Source:HGNC Symbol;Acc:HGNC:24338] | 22 | 1.098 | 0.0193 | Yes |
| 2 | COG5 | component of oligomeric golgi complex 5 [Source:HGNC Symbol;Acc:HGNC:14857] | 38 | 1.012 | 0.0376 | Yes |
| 3 | B3GALNT1 | "beta-1,3-N-acetylgalactosaminyltransferase 1 (globoside blood group) [Source:HGNC Symbol;Acc:HGNC:918]" | 101 | 0.914 | 0.0502 | Yes |
| 4 | TMEM258 | transmembrane protein 258 [Source:HGNC Symbol;Acc:HGNC:1164] | 123 | 0.884 | 0.0656 | Yes |
| 5 | ST8SIA1 | "ST8 alpha-N-acetyl-neuraminide alpha-2,8-sialyltransferase 1 [Source:HGNC Symbol;Acc:HGNC:10869]" | 133 | 0.873 | 0.0816 | Yes |
| 6 | GALNT11 | polypeptide N-acetylgalactosaminyltransferase 11 [Source:HGNC Symbol;Acc:HGNC:19875] | 164 | 0.845 | 0.0955 | Yes |
| 7 | EXT2 | exostosin glycosyltransferase 2 [Source:HGNC Symbol;Acc:HGNC:3513] | 170 | 0.838 | 0.1111 | Yes |
| 8 | MGAT4C | MGAT4 family member C [Source:HGNC Symbol;Acc:HGNC:30871] | 234 | 0.796 | 0.1215 | Yes |
| 9 | CLN5 | CLN5 intracellular trafficking protein [Source:HGNC Symbol;Acc:HGNC:2076] | 248 | 0.785 | 0.1355 | Yes |
| 10 | B4GAT1 | "beta-1,4-glucuronyltransferase 1 [Source:HGNC Symbol;Acc:HGNC:15685]" | 282 | 0.764 | 0.1476 | Yes |
| 11 | EXTL2 | exostosin like glycosyltransferase 2 [Source:HGNC Symbol;Acc:HGNC:3516] | 357 | 0.722 | 0.1556 | Yes |
| 12 | PSEN1 | presenilin 1 [Source:HGNC Symbol;Acc:HGNC:9508] | 425 | 0.695 | 0.1637 | Yes |
| 13 | DDOST | dolichyl-diphosphooligosaccharide--protein glycosyltransferase non-catalytic subunit [Source:HGNC Symbol;Acc:HGNC:2728] | 428 | 0.695 | 0.1769 | Yes |
| 14 | MGAT4A | "alpha-1,3-mannosyl-glycoprotein 4-beta-N-acetylglucosaminyltransferase A [Source:HGNC Symbol;Acc:HGNC:7047]" | 657 | 0.604 | 0.1705 | Yes |
| 15 | DOLK | dolichol kinase [Source:HGNC Symbol;Acc:HGNC:23406] | 683 | 0.598 | 0.1800 | Yes |
| 16 | ALG6 | "ALG6 alpha-1,3-glucosyltransferase [Source:HGNC Symbol;Acc:HGNC:23157]" | 696 | 0.595 | 0.1905 | Yes |
| 17 | DAD1 | defender against cell death 1 [Source:HGNC Symbol;Acc:HGNC:2664] | 737 | 0.585 | 0.1986 | Yes |
| 18 | ALG8 | "ALG8 alpha-1,3-glucosyltransferase [Source:HGNC Symbol;Acc:HGNC:23161]" | 900 | 0.543 | 0.1963 | Yes |
| 19 | ALG5 | ALG5 dolichyl-phosphate beta-glucosyltransferase [Source:HGNC Symbol;Acc:HGNC:20266] | 945 | 0.529 | 0.2030 | Yes |
| 20 | B3GALT4 | "beta-1,3-galactosyltransferase 4 [Source:HGNC Symbol;Acc:HGNC:919]" | 962 | 0.526 | 0.2118 | Yes |
| 21 | COG4 | component of oligomeric golgi complex 4 [Source:HGNC Symbol;Acc:HGNC:18620] | 966 | 0.525 | 0.2217 | Yes |
| 22 | PIGV | phosphatidylinositol glycan anchor biosynthesis class V [Source:HGNC Symbol;Acc:HGNC:26031] | 1064 | 0.503 | 0.2237 | Yes |
| 23 | MAN2A1 | mannosidase alpha class 2A member 1 [Source:HGNC Symbol;Acc:HGNC:6824] | 1069 | 0.501 | 0.2330 | Yes |
| 24 | C1GALT1 | "core 1 synthase, glycoprotein-N-acetylgalactosamine 3-beta-galactosyltransferase 1 [Source:HGNC Symbol;Acc:HGNC:24337]" | 1110 | 0.494 | 0.2393 | Yes |
| 25 | GALNT10 | polypeptide N-acetylgalactosaminyltransferase 10 [Source:HGNC Symbol;Acc:HGNC:19873] | 1173 | 0.481 | 0.2437 | Yes |
| 26 | MAN1C1 | mannosidase alpha class 1C member 1 [Source:HGNC Symbol;Acc:HGNC:19080] | 1275 | 0.463 | 0.2446 | Yes |
| 27 | RPN2 | ribophorin II [Source:HGNC Symbol;Acc:HGNC:10382] | 1294 | 0.459 | 0.2520 | Yes |
| 28 | B4GALT7 | "beta-1,4-galactosyltransferase 7 [Source:HGNC Symbol;Acc:HGNC:930]" | 1411 | 0.442 | 0.2514 | Yes |
| 29 | B3GNT2 | "UDP-GlcNAc:betaGal beta-1,3-N-acetylglucosaminyltransferase 2 [Source:HGNC Symbol;Acc:HGNC:15629]" | 1427 | 0.439 | 0.2586 | Yes |
| 30 | UBE2J1 | ubiquitin conjugating enzyme E2 J1 [Source:HGNC Symbol;Acc:HGNC:17598] | 1433 | 0.438 | 0.2666 | Yes |
| 31 | ALG9 | "ALG9 alpha-1,2-mannosyltransferase [Source:HGNC Symbol;Acc:HGNC:15672]" | 1487 | 0.431 | 0.2708 | Yes |
| 32 | ABO | "ABO, alpha 1-3-N-acetylgalactosaminyltransferase and alpha 1-3-galactosyltransferase [Source:HGNC Symbol;Acc:HGNC:79]" | 1495 | 0.429 | 0.2784 | Yes |
| 33 | B4GALT6 | "beta-1,4-galactosyltransferase 6 [Source:HGNC Symbol;Acc:HGNC:929]" | 1500 | 0.429 | 0.2864 | Yes |
| 34 | FUT8 | fucosyltransferase 8 [Source:HGNC Symbol;Acc:HGNC:4019] | 1565 | 0.418 | 0.2894 | Yes |
| 35 | NAGPA | N-acetylglucosamine-1-phosphodiester alpha-N-acetylglucosaminidase [Source:HGNC Symbol;Acc:HGNC:17378] | 1608 | 0.412 | 0.2940 | Yes |
| 36 | TMEM59 | transmembrane protein 59 [Source:HGNC Symbol;Acc:HGNC:1239] | 1644 | 0.407 | 0.2990 | Yes |
| 37 | GCNT3 | "glucosaminyl (N-acetyl) transferase 3, mucin type [Source:HGNC Symbol;Acc:HGNC:4205]" | 1683 | 0.401 | 0.3037 | Yes |
| 38 | COG7 | component of oligomeric golgi complex 7 [Source:HGNC Symbol;Acc:HGNC:18622] | 1691 | 0.400 | 0.3109 | Yes |
| 39 | ST8SIA4 | "ST8 alpha-N-acetyl-neuraminide alpha-2,8-sialyltransferase 4 [Source:HGNC Symbol;Acc:HGNC:10871]" | 1850 | 0.379 | 0.3057 | No |
| 40 | GCNT1 | glucosaminyl (N-acetyl) transferase 1 [Source:HGNC Symbol;Acc:HGNC:4203] | 2035 | 0.357 | 0.2981 | No |
| 41 | FUT4 | fucosyltransferase 4 [Source:HGNC Symbol;Acc:HGNC:4015] | 2318 | 0.324 | 0.2822 | No |
| 42 | FUT7 | fucosyltransferase 7 [Source:HGNC Symbol;Acc:HGNC:4018] | 2343 | 0.322 | 0.2865 | No |
| 43 | MLEC | malectin [Source:HGNC Symbol;Acc:HGNC:28973] | 2436 | 0.310 | 0.2852 | No |
| 44 | SLC35C2 | solute carrier family 35 member C2 [Source:HGNC Symbol;Acc:HGNC:17117] | 2563 | 0.295 | 0.2810 | No |
| 45 | IL15 | interleukin 15 [Source:HGNC Symbol;Acc:HGNC:5977] | 2599 | 0.291 | 0.2838 | No |
| 46 | DPM3 | "dolichyl-phosphate mannosyltransferase subunit 3, regulatory [Source:HGNC Symbol;Acc:HGNC:3007]" | 2618 | 0.289 | 0.2879 | No |
| 47 | ST6GALNAC5 | "ST6 N-acetylgalactosaminide alpha-2,6-sialyltransferase 5 [Source:HGNC Symbol;Acc:HGNC:19342]" | 2641 | 0.286 | 0.2917 | No |
| 48 | PIGB | phosphatidylinositol glycan anchor biosynthesis class B [Source:HGNC Symbol;Acc:HGNC:8959] | 2663 | 0.284 | 0.2955 | No |
| 49 | B3GALT2 | "beta-1,3-galactosyltransferase 2 [Source:HGNC Symbol;Acc:HGNC:917]" | 2883 | 0.262 | 0.2833 | No |
| 50 | GALNT7 | polypeptide N-acetylgalactosaminyltransferase 7 [Source:HGNC Symbol;Acc:HGNC:4129] | 2954 | 0.255 | 0.2827 | No |
| 51 | B3GAT3 | "beta-1,3-glucuronyltransferase 3 [Source:HGNC Symbol;Acc:HGNC:923]" | 2987 | 0.251 | 0.2850 | No |
| 52 | PLOD1 | "procollagen-lysine,2-oxoglutarate 5-dioxygenase 1 [Source:HGNC Symbol;Acc:HGNC:9081]" | 3155 | 0.235 | 0.2764 | No |
| 53 | POGLUT2 | protein O-glucosyltransferase 2 [Source:HGNC Symbol;Acc:HGNC:19350] | 3238 | 0.227 | 0.2743 | No |
| 54 | EXT1 | exostosin glycosyltransferase 1 [Source:HGNC Symbol;Acc:HGNC:3512] | 3321 | 0.219 | 0.2721 | No |
| 55 | GBA | glucosylceramidase beta [Source:HGNC Symbol;Acc:HGNC:4177] | 3327 | 0.219 | 0.2759 | No |
| 56 | ST3GAL6 | "ST3 beta-galactoside alpha-2,3-sialyltransferase 6 [Source:HGNC Symbol;Acc:HGNC:18080]" | 3339 | 0.218 | 0.2792 | No |
| 57 | B4GALT4 | "beta-1,4-galactosyltransferase 4 [Source:HGNC Symbol;Acc:HGNC:927]" | 3407 | 0.211 | 0.2780 | No |
| 58 | MAN1B1 | mannosidase alpha class 1B member 1 [Source:HGNC Symbol;Acc:HGNC:6823] | 3447 | 0.207 | 0.2789 | No |
| 59 | ST6GAL1 | "ST6 beta-galactoside alpha-2,6-sialyltransferase 1 [Source:HGNC Symbol;Acc:HGNC:10860]" | 3502 | 0.202 | 0.2785 | No |
| 60 | PLOD3 | "procollagen-lysine,2-oxoglutarate 5-dioxygenase 3 [Source:HGNC Symbol;Acc:HGNC:9083]" | 3614 | 0.192 | 0.2735 | No |
| 61 | POMGNT1 | "protein O-linked mannose N-acetylglucosaminyltransferase 1 (beta 1,2-) [Source:HGNC Symbol;Acc:HGNC:19139]" | 3641 | 0.191 | 0.2751 | No |
| 62 | ST3GAL5 | "ST3 beta-galactoside alpha-2,3-sialyltransferase 5 [Source:HGNC Symbol;Acc:HGNC:10872]" | 3667 | 0.188 | 0.2767 | No |
| 63 | ALG12 | "ALG12 alpha-1,6-mannosyltransferase [Source:HGNC Symbol;Acc:HGNC:19358]" | 3723 | 0.183 | 0.2759 | No |
| 64 | COG2 | component of oligomeric golgi complex 2 [Source:HGNC Symbol;Acc:HGNC:6546] | 3767 | 0.180 | 0.2760 | No |
| 65 | GCNT4 | glucosaminyl (N-acetyl) transferase 4 [Source:HGNC Symbol;Acc:HGNC:17973] | 3858 | 0.169 | 0.2722 | No |
| 66 | DPY19L1 | dpy-19 like C-mannosyltransferase 1 [Source:HGNC Symbol;Acc:HGNC:22205] | 3875 | 0.167 | 0.2741 | No |
| 67 | MAGT1 | magnesium transporter 1 [Source:HGNC Symbol;Acc:HGNC:28880] | 3920 | 0.163 | 0.2738 | No |
| 68 | TUSC3 | tumor suppressor candidate 3 [Source:HGNC Symbol;Acc:HGNC:30242] | 3940 | 0.161 | 0.2754 | No |
| 69 | TRAK1 | trafficking kinesin protein 1 [Source:HGNC Symbol;Acc:HGNC:29947] | 3964 | 0.159 | 0.2766 | No |
| 70 | ALG3 | "ALG3 alpha-1,3- mannosyltransferase [Source:HGNC Symbol;Acc:HGNC:23056]" | 4096 | 0.146 | 0.2692 | No |
| 71 | ST8SIA2 | "ST8 alpha-N-acetyl-neuraminide alpha-2,8-sialyltransferase 2 [Source:HGNC Symbol;Acc:HGNC:10870]" | 4153 | 0.142 | 0.2675 | No |
| 72 | RAMP1 | receptor activity modifying protein 1 [Source:HGNC Symbol;Acc:HGNC:9843] | 4201 | 0.138 | 0.2664 | No |
| 73 | PMM1 | phosphomannomutase 1 [Source:HGNC Symbol;Acc:HGNC:9114] | 4238 | 0.134 | 0.2662 | No |
| 74 | MAN1A2 | mannosidase alpha class 1A member 2 [Source:HGNC Symbol;Acc:HGNC:6822] | 4272 | 0.131 | 0.2661 | No |
| 75 | DHDDS | dehydrodolichyl diphosphate synthase subunit [Source:HGNC Symbol;Acc:HGNC:20603] | 4356 | 0.124 | 0.2620 | No |
| 76 | STT3A | STT3 oligosaccharyltransferase complex catalytic subunit A [Source:HGNC Symbol;Acc:HGNC:6172] | 4618 | 0.103 | 0.2434 | No |
| 77 | POMK | protein O-mannose kinase [Source:HGNC Symbol;Acc:HGNC:26267] | 4680 | 0.098 | 0.2405 | No |
| 78 | B3GAT1 | "beta-1,3-glucuronyltransferase 1 [Source:HGNC Symbol;Acc:HGNC:921]" | 4715 | 0.095 | 0.2397 | No |
| 79 | DPM2 | "dolichyl-phosphate mannosyltransferase subunit 2, regulatory [Source:HGNC Symbol;Acc:HGNC:3006]" | 4862 | 0.083 | 0.2298 | No |
| 80 | POFUT1 | protein O-fucosyltransferase 1 [Source:HGNC Symbol;Acc:HGNC:14988] | 4910 | 0.079 | 0.2276 | No |
| 81 | MGAT5 | "alpha-1,6-mannosylglycoprotein 6-beta-N-acetylglucosaminyltransferase [Source:HGNC Symbol;Acc:HGNC:7049]" | 5086 | 0.066 | 0.2151 | No |
| 82 | B3GALT5 | "beta-1,3-galactosyltransferase 5 [Source:HGNC Symbol;Acc:HGNC:920]" | 5132 | 0.061 | 0.2128 | No |
| 83 | DPY19L4 | dpy-19 like 4 [Source:HGNC Symbol;Acc:HGNC:27829] | 5133 | 0.061 | 0.2139 | No |
| 84 | TRIP11 | thyroid hormone receptor interactor 11 [Source:HGNC Symbol;Acc:HGNC:12305] | 5233 | 0.054 | 0.2072 | No |
| 85 | PGM3 | phosphoglucomutase 3 [Source:HGNC Symbol;Acc:HGNC:8907] | 5290 | 0.049 | 0.2037 | No |
| 86 | MGAT2 | "alpha-1,6-mannosyl-glycoprotein 2-beta-N-acetylglucosaminyltransferase [Source:HGNC Symbol;Acc:HGNC:7045]" | 5317 | 0.046 | 0.2026 | No |
| 87 | ABCA2 | ATP binding cassette subfamily A member 2 [Source:HGNC Symbol;Acc:HGNC:32] | 5349 | 0.044 | 0.2010 | No |
| 88 | DOLPP1 | dolichyldiphosphatase 1 [Source:HGNC Symbol;Acc:HGNC:29565] | 5369 | 0.043 | 0.2003 | No |
| 89 | MGAT1 | "alpha-1,3-mannosyl-glycoprotein 2-beta-N-acetylglucosaminyltransferase [Source:HGNC Symbol;Acc:HGNC:7044]" | 5407 | 0.040 | 0.1982 | No |
| 90 | NPC1 | NPC intracellular cholesterol transporter 1 [Source:HGNC Symbol;Acc:HGNC:7897] | 5480 | 0.034 | 0.1932 | No |
| 91 | GALNT4 | polypeptide N-acetylgalactosaminyltransferase 4 [Source:HGNC Symbol;Acc:HGNC:4126] | 5610 | 0.023 | 0.1835 | No |
| 92 | GFPT1 | glutamine--fructose-6-phosphate transaminase 1 [Source:HGNC Symbol;Acc:HGNC:4241] | 5847 | 0.005 | 0.1650 | No |
| 93 | GFPT2 | glutamine-fructose-6-phosphate transaminase 2 [Source:HGNC Symbol;Acc:HGNC:4242] | 5849 | 0.005 | 0.1651 | No |
| 94 | B3GALT1 | "beta-1,3-galactosyltransferase 1 [Source:HGNC Symbol;Acc:HGNC:916]" | 5864 | 0.004 | 0.1640 | No |
| 95 | GALNT6 | polypeptide N-acetylgalactosaminyltransferase 6 [Source:HGNC Symbol;Acc:HGNC:4128] | 5915 | 0.001 | 0.1601 | No |
| 96 | MGAT4B | "alpha-1,3-mannosyl-glycoprotein 4-beta-N-acetylglucosaminyltransferase B [Source:HGNC Symbol;Acc:HGNC:7048]" | 5956 | -0.002 | 0.1570 | No |
| 97 | RXYLT1 | ribitol xylosyltransferase 1 [Source:HGNC Symbol;Acc:HGNC:13530] | 6030 | -0.007 | 0.1514 | No |
| 98 | EDEM3 | ER degradation enhancing alpha-mannosidase like protein 3 [Source:HGNC Symbol;Acc:HGNC:16787] | 6073 | -0.010 | 0.1483 | No |
| 99 | EXTL1 | exostosin like glycosyltransferase 1 [Source:HGNC Symbol;Acc:HGNC:3515] | 6193 | -0.020 | 0.1394 | No |
| 100 | GALNT1 | polypeptide N-acetylgalactosaminyltransferase 1 [Source:HGNC Symbol;Acc:HGNC:4123] | 6217 | -0.022 | 0.1380 | No |
| 101 | NUS1 | NUS1 dehydrodolichyl diphosphate synthase subunit [Source:HGNC Symbol;Acc:HGNC:21042] | 6415 | -0.037 | 0.1232 | No |
| 102 | SLC39A8 | solute carrier family 39 member 8 [Source:HGNC Symbol;Acc:HGNC:20862] | 6450 | -0.039 | 0.1213 | No |
| 103 | GORASP1 | golgi reassembly stacking protein 1 [Source:HGNC Symbol;Acc:HGNC:16769] | 6526 | -0.044 | 0.1162 | No |
| 104 | ST8SIA5 | "ST8 alpha-N-acetyl-neuraminide alpha-2,8-sialyltransferase 5 [Source:HGNC Symbol;Acc:HGNC:17827]" | 6565 | -0.047 | 0.1141 | No |
| 105 | RPN1 | ribophorin I [Source:HGNC Symbol;Acc:HGNC:10381] | 6618 | -0.051 | 0.1110 | No |
| 106 | POMT1 | protein O-mannosyltransferase 1 [Source:HGNC Symbol;Acc:HGNC:9202] | 6652 | -0.053 | 0.1095 | No |
| 107 | GRM7 | glutamate metabotropic receptor 7 [Source:HGNC Symbol;Acc:HGNC:4599] | 6694 | -0.055 | 0.1073 | No |
| 108 | GALNT14 | polypeptide N-acetylgalactosaminyltransferase 14 [Source:HGNC Symbol;Acc:HGNC:22946] | 6762 | -0.060 | 0.1032 | No |
| 109 | FKRP | fukutin related protein [Source:HGNC Symbol;Acc:HGNC:17997] | 7065 | -0.085 | 0.0811 | No |
| 110 | OGA | O-GlcNAcase [Source:HGNC Symbol;Acc:HGNC:7056] | 7086 | -0.086 | 0.0812 | No |
| 111 | LARGE1 | LARGE xylosyl- and glucuronyltransferase 1 [Source:HGNC Symbol;Acc:HGNC:6511] | 7164 | -0.093 | 0.0769 | No |
| 112 | SRD5A3 | steroid 5 alpha-reductase 3 [Source:HGNC Symbol;Acc:HGNC:25812] | 7325 | -0.104 | 0.0664 | No |
| 113 | DPAGT1 | dolichyl-phosphate N-acetylglucosaminephosphotransferase 1 [Source:HGNC Symbol;Acc:HGNC:2995] | 7452 | -0.115 | 0.0587 | No |
| 114 | GALNT2 | polypeptide N-acetylgalactosaminyltransferase 2 [Source:HGNC Symbol;Acc:HGNC:4124] | 7453 | -0.115 | 0.0609 | No |
| 115 | NANS | N-acetylneuraminate synthase [Source:HGNC Symbol;Acc:HGNC:19237] | 7530 | -0.121 | 0.0572 | No |
| 116 | DPM1 | "dolichyl-phosphate mannosyltransferase subunit 1, catalytic [Source:HGNC Symbol;Acc:HGNC:3005]" | 7723 | -0.135 | 0.0447 | No |
| 117 | B4GALNT1 | "beta-1,4-N-acetyl-galactosaminyltransferase 1 [Source:HGNC Symbol;Acc:HGNC:4117]" | 7799 | -0.141 | 0.0415 | No |
| 118 | GALNT8 | polypeptide N-acetylgalactosaminyltransferase 8 [Source:HGNC Symbol;Acc:HGNC:4130] | 8124 | -0.164 | 0.0193 | No |
| 119 | MPDU1 | mannose-P-dolichol utilization defect 1 [Source:HGNC Symbol;Acc:HGNC:7207] | 8330 | -0.180 | 0.0066 | No |
| 120 | B3GNT3 | "UDP-GlcNAc:betaGal beta-1,3-N-acetylglucosaminyltransferase 3 [Source:HGNC Symbol;Acc:HGNC:13528]" | 8361 | -0.183 | 0.0078 | No |
| 121 | GAL3ST1 | galactose-3-O-sulfotransferase 1 [Source:HGNC Symbol;Acc:HGNC:24240] | 8378 | -0.184 | 0.0100 | No |
| 122 | UGGT2 | UDP-glucose glycoprotein glucosyltransferase 2 [Source:HGNC Symbol;Acc:HGNC:15664] | 8568 | -0.199 | -0.0010 | No |
| 123 | GCNT2 | glucosaminyl (N-acetyl) transferase 2 (I blood group) [Source:HGNC Symbol;Acc:HGNC:4204] | 8619 | -0.202 | -0.0010 | No |
| 124 | VEGFB | vascular endothelial growth factor B [Source:HGNC Symbol;Acc:HGNC:12681] | 8673 | -0.206 | -0.0012 | No |
| 125 | ST6GALNAC2 | "ST6 N-acetylgalactosaminide alpha-2,6-sialyltransferase 2 [Source:HGNC Symbol;Acc:HGNC:10867]" | 8870 | -0.222 | -0.0124 | No |
| 126 | FUT5 | fucosyltransferase 5 [Source:HGNC Symbol;Acc:HGNC:4016] | 8898 | -0.225 | -0.0102 | No |
| 127 | MGAT3 | "beta-1,4-mannosyl-glycoprotein 4-beta-N-acetylglucosaminyltransferase [Source:HGNC Symbol;Acc:HGNC:7046]" | 8920 | -0.226 | -0.0075 | No |
| 128 | PIGZ | phosphatidylinositol glycan anchor biosynthesis class Z [Source:HGNC Symbol;Acc:HGNC:30596] | 9047 | -0.235 | -0.0129 | No |
| 129 | B4GALT5 | "beta-1,4-galactosyltransferase 5 [Source:HGNC Symbol;Acc:HGNC:928]" | 9051 | -0.236 | -0.0086 | No |
| 130 | POMT2 | protein O-mannosyltransferase 2 [Source:HGNC Symbol;Acc:HGNC:19743] | 9216 | -0.249 | -0.0167 | No |
| 131 | SERP1 | stress associated endoplasmic reticulum protein 1 [Source:HGNC Symbol;Acc:HGNC:10759] | 9343 | -0.259 | -0.0216 | No |
| 132 | PMM2 | phosphomannomutase 2 [Source:HGNC Symbol;Acc:HGNC:9115] | 9408 | -0.265 | -0.0216 | No |
| 133 | ENTPD5 | ectonucleoside triphosphate diphosphohydrolase 5 (inactive) [Source:HGNC Symbol;Acc:HGNC:3367] | 9414 | -0.265 | -0.0169 | No |
| 134 | FUT9 | fucosyltransferase 9 [Source:HGNC Symbol;Acc:HGNC:4020] | 9433 | -0.268 | -0.0132 | No |
| 135 | B3GNT4 | "UDP-GlcNAc:betaGal beta-1,3-N-acetylglucosaminyltransferase 4 [Source:HGNC Symbol;Acc:HGNC:15683]" | 9500 | -0.274 | -0.0131 | No |
| 136 | UGGT1 | UDP-glucose glycoprotein glucosyltransferase 1 [Source:HGNC Symbol;Acc:HGNC:15663] | 9516 | -0.275 | -0.0090 | No |
| 137 | FKTN | fukutin [Source:HGNC Symbol;Acc:HGNC:3622] | 9745 | -0.296 | -0.0212 | No |
| 138 | POGLUT1 | protein O-glucosyltransferase 1 [Source:HGNC Symbol;Acc:HGNC:22954] | 9794 | -0.301 | -0.0192 | No |
| 139 | FUT3 | fucosyltransferase 3 (Lewis blood group) [Source:HGNC Symbol;Acc:HGNC:4014] | 9890 | -0.311 | -0.0207 | No |
| 140 | CHST4 | carbohydrate sulfotransferase 4 [Source:HGNC Symbol;Acc:HGNC:1972] | 9941 | -0.315 | -0.0186 | No |
| 141 | TET3 | tet methylcytosine dioxygenase 3 [Source:HGNC Symbol;Acc:HGNC:28313] | 9962 | -0.317 | -0.0141 | No |
| 142 | ST8SIA3 | "ST8 alpha-N-acetyl-neuraminide alpha-2,8-sialyltransferase 3 [Source:HGNC Symbol;Acc:HGNC:14269]" | 10046 | -0.326 | -0.0143 | No |
| 143 | PLOD2 | "procollagen-lysine,2-oxoglutarate 5-dioxygenase 2 [Source:HGNC Symbol;Acc:HGNC:9082]" | 10086 | -0.330 | -0.0111 | No |
| 144 | EOGT | EGF domain specific O-linked N-acetylglucosamine transferase [Source:HGNC Symbol;Acc:HGNC:28526] | 10174 | -0.338 | -0.0114 | No |
| 145 | MAN1A1 | mannosidase alpha class 1A member 1 [Source:HGNC Symbol;Acc:HGNC:6821] | 10207 | -0.341 | -0.0074 | No |
| 146 | GOLGA2 | golgin A2 [Source:HGNC Symbol;Acc:HGNC:4425] | 10285 | -0.349 | -0.0067 | No |
| 147 | GALNT12 | polypeptide N-acetylgalactosaminyltransferase 12 [Source:HGNC Symbol;Acc:HGNC:19877] | 10414 | -0.362 | -0.0098 | No |
| 148 | ARFGEF1 | ADP ribosylation factor guanine nucleotide exchange factor 1 [Source:HGNC Symbol;Acc:HGNC:15772] | 10477 | -0.371 | -0.0076 | No |
| 149 | B4GALT2 | "beta-1,4-galactosyltransferase 2 [Source:HGNC Symbol;Acc:HGNC:925]" | 10490 | -0.372 | -0.0014 | No |
| 150 | MOGS | mannosyl-oligosaccharide glucosidase [Source:HGNC Symbol;Acc:HGNC:24862] | 10630 | -0.386 | -0.0049 | No |
| 151 | ST3GAL4 | "ST3 beta-galactoside alpha-2,3-sialyltransferase 4 [Source:HGNC Symbol;Acc:HGNC:10864]" | 10688 | -0.391 | -0.0019 | No |
| 152 | CHP1 | calcineurin like EF-hand protein 1 [Source:HGNC Symbol;Acc:HGNC:17433] | 10940 | -0.423 | -0.0135 | No |
| 153 | GALNT3 | polypeptide N-acetylgalactosaminyltransferase 3 [Source:HGNC Symbol;Acc:HGNC:4125] | 11062 | -0.440 | -0.0145 | No |
| 154 | FUT1 | fucosyltransferase 1 (H blood group) [Source:HGNC Symbol;Acc:HGNC:4012] | 11086 | -0.442 | -0.0079 | No |
| 155 | ST6GALNAC4 | "ST6 N-acetylgalactosaminide alpha-2,6-sialyltransferase 4 [Source:HGNC Symbol;Acc:HGNC:17846]" | 11164 | -0.452 | -0.0052 | No |
| 156 | SLC35C1 | solute carrier family 35 member C1 [Source:HGNC Symbol;Acc:HGNC:20197] | 11286 | -0.470 | -0.0057 | No |
| 157 | FUT2 | fucosyltransferase 2 [Source:HGNC Symbol;Acc:HGNC:4013] | 11459 | -0.497 | -0.0097 | No |
| 158 | B4GALT1 | "beta-1,4-galactosyltransferase 1 [Source:HGNC Symbol;Acc:HGNC:924]" | 11530 | -0.505 | -0.0055 | No |
| 159 | A4GNT | "alpha-1,4-N-acetylglucosaminyltransferase [Source:HGNC Symbol;Acc:HGNC:17968]" | 11568 | -0.510 | 0.0014 | No |
| 160 | TMEM165 | transmembrane protein 165 [Source:HGNC Symbol;Acc:HGNC:30760] | 11578 | -0.512 | 0.0105 | No |
| 161 | ST3GAL2 | "ST3 beta-galactoside alpha-2,3-sialyltransferase 2 [Source:HGNC Symbol;Acc:HGNC:10863]" | 11721 | -0.536 | 0.0096 | No |
| 162 | POFUT2 | protein O-fucosyltransferase 2 [Source:HGNC Symbol;Acc:HGNC:14683] | 11725 | -0.537 | 0.0197 | No |
| 163 | B4GALT3 | "beta-1,4-galactosyltransferase 3 [Source:HGNC Symbol;Acc:HGNC:926]" | 11748 | -0.540 | 0.0283 | No |
| 164 | MAN2A2 | mannosidase alpha class 2A member 2 [Source:HGNC Symbol;Acc:HGNC:6825] | 11772 | -0.545 | 0.0370 | No |
| 165 | FUT6 | fucosyltransferase 6 [Source:HGNC Symbol;Acc:HGNC:4017] | 12067 | -0.612 | 0.0256 | No |
| 166 | ST3GAL1 | "ST3 beta-galactoside alpha-2,3-sialyltransferase 1 [Source:HGNC Symbol;Acc:HGNC:10862]" | 12092 | -0.619 | 0.0356 | No |
| 167 | OGT | O-linked N-acetylglucosamine (GlcNAc) transferase [Source:HGNC Symbol;Acc:HGNC:8127] | 12440 | -0.721 | 0.0222 | No |
| 168 | EXTL3 | exostosin like glycosyltransferase 3 [Source:HGNC Symbol;Acc:HGNC:3518] | 12442 | -0.722 | 0.0360 | No |
Table: GSEA details [plain text format]

  

Fig 2: GOBP\_GLYCOSYLATION      
 Blue-Pink O' Gram in the Space of the Analyzed GeneSet

  

Fig 3: GOBP\_GLYCOSYLATION: Random ES distribution      
 Gene set null distribution of ES for **GOBP\_GLYCOSYLATION**
